# Tracking late Pleistocene Neandertals on the Iberian coast

**DOI:** 10.1038/s41598-021-83413-8

**Published:** 2021-03-11

**Authors:** Eduardo Mayoral, Ignacio Díaz-Martínez, Jéremy Duveau, Ana Santos, Antonio Rodríguez Ramírez, Juan A. Morales, Luis A. Morales, Ricardo Díaz-Delgado

**Affiliations:** 1 Departamento de Ciencias de la Tierra, Facultad de Ciencias Experimentales, Campus de el Carmen, Universidad de Huelva, Huelva, Spain; 2 CCTH-Centro de Investigación Científico Tecnológico, Universidad de Huelva, Huelva, Spain; 3 Universidad Nacional de Río Negro-IIPG, General Roca, Río Negro Argentina; 4 Instituto de Investigación en Paleobiología y Geología (IIPG), CONICET, General Roca, Río Negro Argentina; 5 Histoire Naturelle de l’Homme Préhistorique, CNRS, Muséum National d’Histoire Naturelle, Université Perpignan Via Domitia, Paris, France; 6 Al Futuro Arquitectura, Huelva, Spain; 7 Estación Biológica de Doñana-CSIC, Seville, Spain

**Keywords:** Ecology, Evolution

## Abstract

Here, we report the recent discovery of 87 Neandertal footprints on the Southwest of the Iberian Peninsula (Doñana shoreline, Spain) located on an upper Pleistocene aeolian littoral setting (about 106 ± 19 kyr). Morphometric comparisons, high resolution digital photogrammetric 3D models and detailed sedimentary analysis have been provided to characterized the footprints and the palaeoenvironment. The footprints were impressed in the shoreline of a hypersaline swamped area related to benthic microbial mats, close to the coastline. They have a rounded heel, a longitudinal arch, relatively short toes, and adducted hallux, and represent the oldest upper Pleistocene record of Neandertal footprints in the world. Among these 87 footprints, 31 are longitudinally complete and measure from 14 to 29 cm. The calculated statures range from 104 to 188 cm, with half of the data between 130 and 150 cm. The wide range of sizes of the footprints suggests the existence of a social group integrated by individuals of different age classes but dominated, however, by non-adult individuals. The footprints, which are outside the flooded area are oriented perpendicular to the shoreline. These 87 footprints reinforce the ecological scenario of Neandertal groups established in coastal areas.

## Introduction

The biological and ethological information of the ancient hominin groups when there are no bone remains, is provided by the study of their fossil footprints, which show us certain "frozen" moments of their existence^1^. Footprints provide invaluable data on the number individuals who made them and on their biological (stature, age, body mass) and biomechanical (posture, gait, speed) characteristics^2–5^. However, and despite important recent discoveries^5–9^, the number of sites with such footprints remains relatively uncommon compared to archaeological or palaeoanthropological sites, especially for those occupied by Neandertals^5^. This is the case of the Iberian Peninsula, where there are several localities with osteological and technological remains of Neandertals^10–12^, but only one potential poorly preserved footprint in the dune complex from Catalán Bay (28,000 years) at Gibraltar^13^.

At the June of 2020 as a consequence of the intense winter storms which, together with the action of spring tidal ranges, left an extensive trampled surface area of just over 6000 m^2^ exposed at Matalascañas (Huelva, SW Spain). This fact has conditioned, and affects, notably, its study, since being inside the intertidal zone, it is daily covered by water and sandy sediments, which from one day to another can cover all or part of the surface exposed for free observation. This leaves open the possibility that additional tracks may be unearthed nearby. Some mammal and bird footprints were identified preliminary there^14,15^. Among all those tracks, several hominin footprints have been found and they are the object of study of this work.

Here we report the discovery of 87 footprints attributed to Neandertals on the site of Matalascañas, on the Southwest of the Iberian Peninsula (Doñana shoreline, Spain). After describing the site and in particular its stratigraphy, we focused on the description of the Neandertal footprints in order to obtain information on the number of individuals that left them and their biological characteristics (age, stature) by using morphometric data.

## The site of Matalascañas

**Figure 1:**
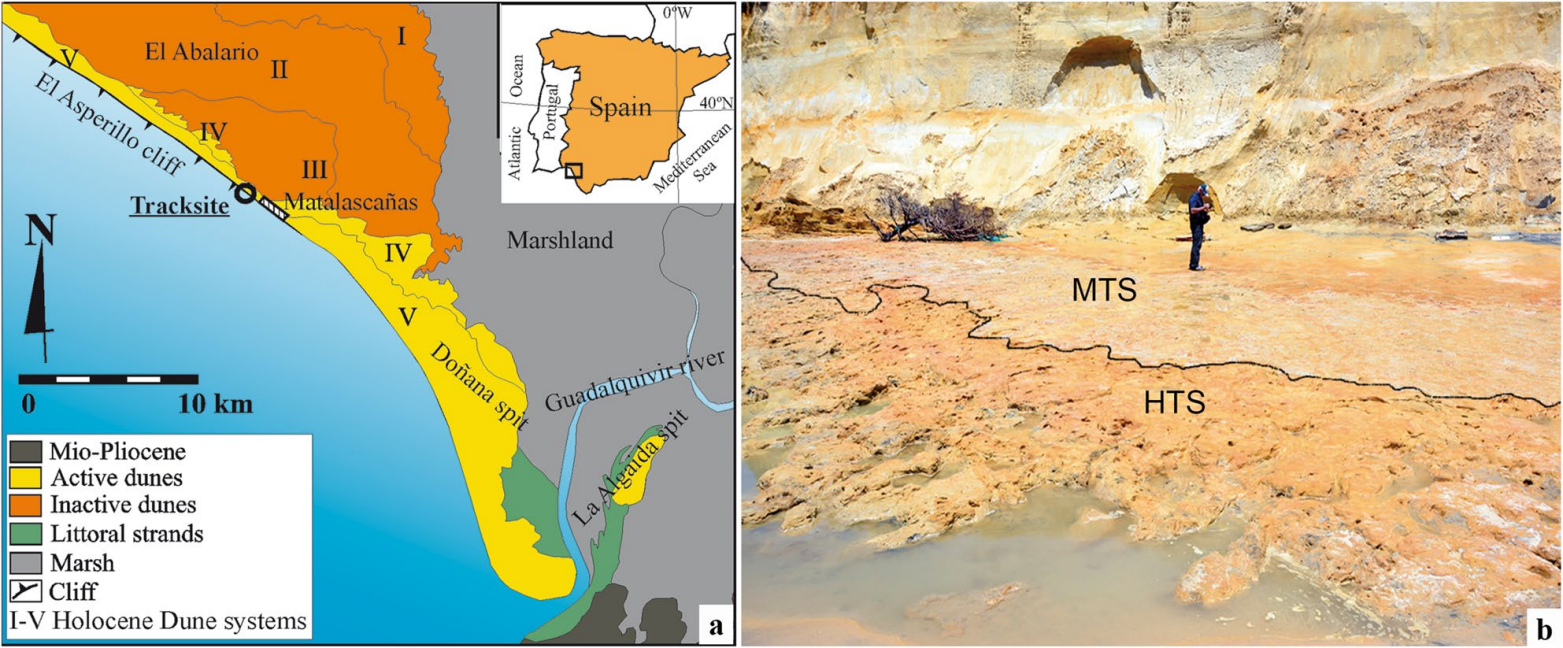
Location of Matalascañas site. (**a**) Geographic and geologic setting. Modified from Rodríguez-Ramírez et al.^16^. (**b**) Field view of the palaeosol PS2. MTS: Matalascañas Trampled Surface^14,15^. HTS: Hominin Trampled Surface (this article).

In the southwest of the Iberian Peninsula is located one of the most important wind systems in Europe, El Abalario, with an area of about 450 km^2^ (Fig. 1a)^16^. This vast dune complex is located in the middle of the Gulf of Cadiz, in the interfluve defined by the valleys of the Guadalquivir and Tinto rivers, stratigraphically supported by the Neogene of the Guadalquivir basin.

Geomorphologically, it has a dome shape, elongated in the NW–SE direction, due to the intense overlapping of the five sequences of Holocene dunes which cover it^17^. Its orography is very gentle, defined by the dune models and the development of important seasonal lagoon systems, only blurred by the more recent dune trains, which reach more than 100 m in height in the area closest to the coast (Asperillo dune system 115 m). The northern and northeastern limits of the Abalario are formed by the Las Madres and Rocina streams, while to the east the dune systems gradually lose height, being buried by the coastal arrows and marshes of the Guadalquivir estuary during the recent Holocene. Towards the SW, the limit of El Abalario is the Atlantic Ocean, which has been intensely eroded by the action of marine dynamics, with values that vary between 0.5 and 1.2 m per year on average^17,18^.

These marine dynamics are characterised by a low energy wave regime, with average waves of less than 1 m, although in winter storms they can often reach 4 m and high percentages of more than 1.5 m^18^. It is a meso-tidal type coast, with a maximum range of about 3 m^19^, which allows the erosion of the backshore when winter storms and high tides coincide^20^.

Matalascañas site is located in the so-called Castilla Beach (37º 0′ 58″ N; – 6º 35′ 0.23″ W) some 45 km SE of the town of Huelva (Spain). In this area, the intense erosion dynamic, together with the sandy, labile and unskilled substratum, has allowed the formation of an important cliff (the Asperillo cliff) of about 30 km in length and an average height of about 20 m, which allows the visualisation of the pre-Holocene sedimentary sequence (Fig. 1b).

**Figure 2:**
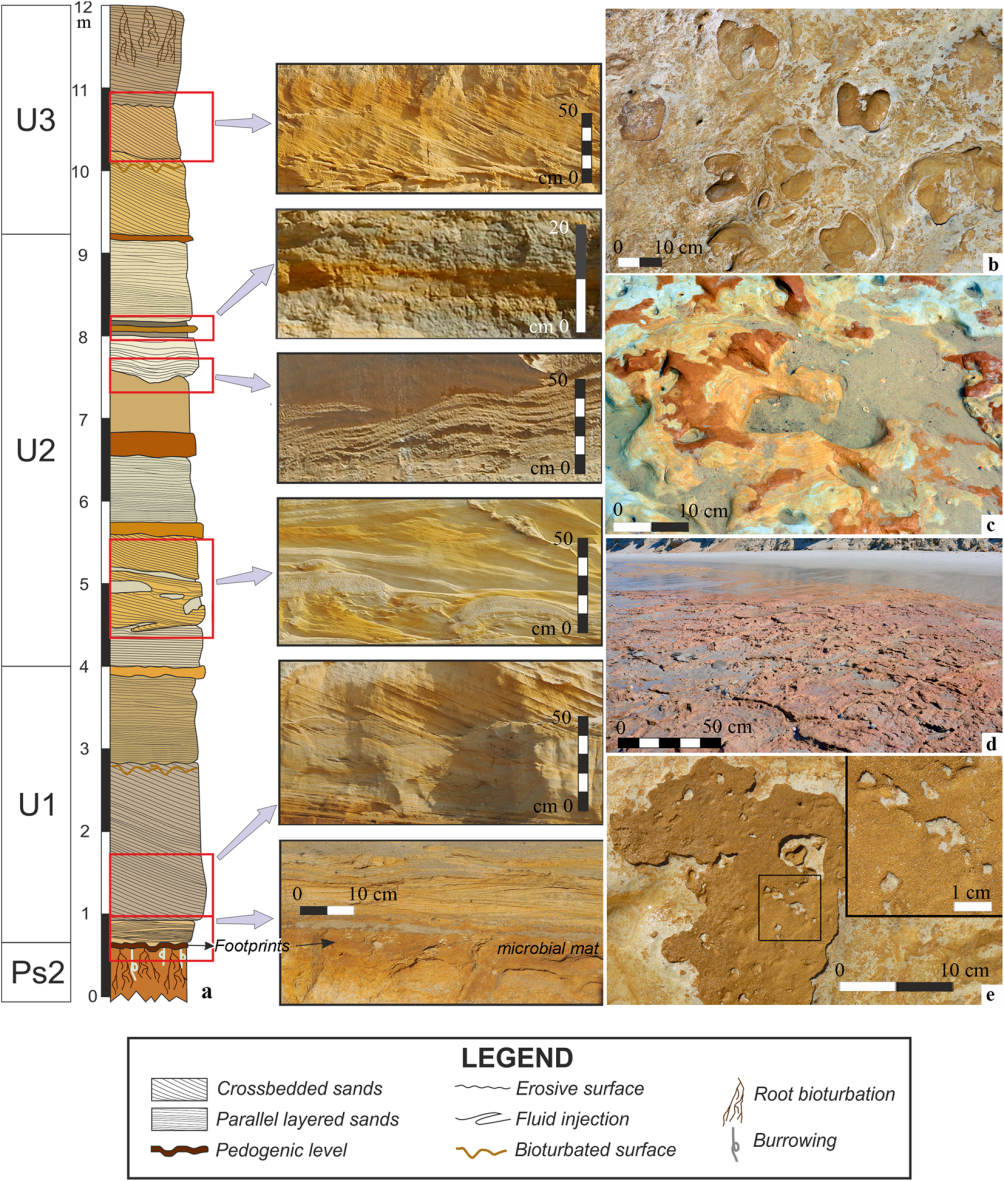
(**a**) Sedimentary log of the Asperillo Cliff in the framework of the units distinguished by Zazo et al.^22^. The right border of the sedimentary bodies in the log indicates the relative changes of the average grain-size. The colors of the bodies represent the approximate real color of the sediments. At the right of the column, pictures illustrate the aspect of some of the sedimentary bodies. (**b**) Detailed view of the MTS with several Artiodactyla tracks. (**c**) Detail of palaeosol Ps2 showing concentric structures and transitions of several colors related to root activity. (**d**) Ferruginized microbial mat overgrown and filled wide polygonal cracks. e. External halite moulds on the ferruginized microbial mat.

The stratigraphic sequence (Fig. 2a) is made up of a series of upper Pleistocene (sensu^21^) aeolian units, defined by Zazo et al.^22^. These Pleistocene units are from bottom to top: Ps2, U1, U2 and U3, on which the Holocene dune sequences (U4–U6) are located. These units are separated by ferruginous palaeosols and levels rich in organic matter (Fig. 2a).

The lowest aeolian unit (U1) lies on a palaeosol (Ps2, sensu^22^) formed by silty sands that represent an irregular surface with an average slope of 2.26º towards the SW. It is precisely on the surface of the discontinuity that separates Ps2 from U1 where coastal erosion has modelled a platform of coastal abrasion, leaving a whole series of fossil tracks and trackways and not on the U1 unit as recently published^14,15^. These series include all hominin footprints (named here as Hominin Trampled Surface, HTS) and the vertebrate tracks (Fig. 2b), composed mainly by Artiodactyla, Elephantidae, Canidae and waterbirds lately named as ‘Matalascañas Trampled Surface’ (MTS)^14,15^.

The basal aeolian Unit 1 is estimates about 106 ± 19 kyr^22^ corresponding to the end of the temperate Last Interglacial (OIS5). Therefore, the track assemblage, including footprints and vertebrate tracks, were made during the upper Pleistocene before the date 106 ± 19 kyr.

**Figure 3:**
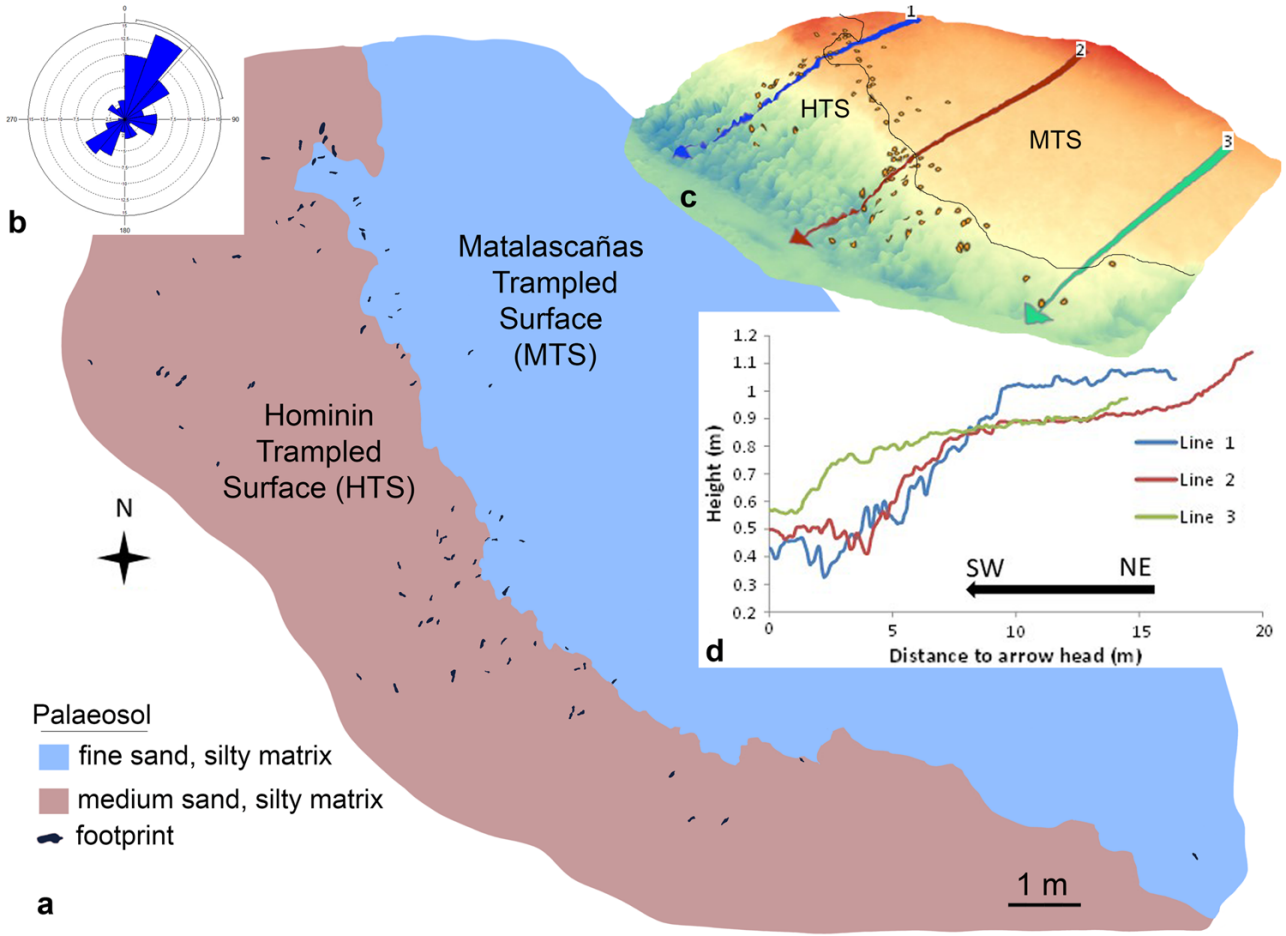
Mapping of the hominin footprints (HTS) and animal tracks (MTS). (**a**) Plan view. (**b**) Wind rose, showing the preferential direction SW-NE of the footprints. (**c**) 3D model of the palaeosol surface with the footprints' location and its relationship with the MTS. (**d**) Elevation profiles according to sections 1 to 3.

The HTS shows hydromorphic processes with vertical elongated segregations about 10–50 cm in diameter (Fig. 2c) suggesting a genetic relation with roots^22^. On this surface, especially in the most humid areas or with a semi-permanent water cover, an extensive microbial mat overgrown and filled wide polygonal cracks that are present throughout most of the area studied (Fig. 2d). Nowadays, the coastal erosion has modelled a wave-cut platform on this muddy layer, exposing the surface with the footprints. A metric-scale level of Holocene sands covers this abrasion platform partially as an ephemeral beach. The dynamics of these sands responds to the wave dimensions. So, the complete sandy layer is dismantled and moved to deeper areas under waves upper 1.5 m high. The hominin footprints are mostly located in an NW–SE band parallel to the current coastline (Fig. 3a,c) and scattered along a band that must have constituted the margin or shore of a large, very shallow flooded area (MTS) where the animal tracks mentioned above are found. In these conditions, the Pleistocene ravinement surface containing the footprints crop out and has conditioned and determines, notably, its study, since it is in a foreshore (intertidal fringe).

## Methods

### Sedimentary analysis

A detailed sedimentary column of the sequence in which the tracked surface is inserted has been recorded. In this column, the units identified by Zazo et al.^22^ are distinguished. Each of these units is integrated by different sand bodies composed of sands of different grain sizes. The particle size distribution of the upper level of Ps2 and other samples of U1 and U2 has been analysed using a standard sieve column (Udden-Wentworth).

### Footprint analyses

#### Footprint recording

Descriptions, measurements, photos and schematic drawings of the footprints were made directly in the field. A low altitude programmed flight was carried out using an Unmanned Aerial Vehicle (UAV) on June the 6th 2020, 12 AM UTC. A multirotor DJI PHANTOM 4+ with a 4 k 20 Mpix RGB CMOS camera collected at an average height of 7 m above the ground a total amount of 360 photographs over the study area (564 m^2^). A photogrammetric orthomosaic was produced using both PIX4DMAPPE and AGISOFT METASHAPE PROFESSIONAL 1.5.1.7618 software. This procedure allowed us to plot the position, measure and photograph each new sediment exposure and its imprints before all were cover by sand in further tides.

Topographic profiles and tridimensional view (Fig. 3c,d) were generated using the DIGITAL SURFACE MODEL (DSM). DSM is a raster file (grid with pixel size = 0.6 m) of the study area where pixel values correspond to topographic height above sea level. Pixel values were interpolated from the Point Cloud composed by triangulation of tie points between drone cameras (pictures) captured in a 50 m height flight. Point cloud contains common geographic coordinates (X, Y, Z) for all the pictures acquired by the UAV.

Moreover, detailed photographs of the best-preserved footprints were used to obtain 3D photogrammetric models with the software AGISOFT METASHAPE PROFESSIONAL 1.5.1.7618. Solid, depth-colour and contour lines images were produced with the software PARAVIEW 4.4.0.

#### Footprint measurements

A total of 87 footprints was measured from the UAV models. The measurements on these models were checked by comparing them with the measurements taken in situ. No significant differences were found.

The footprint length was measured along the longitudinal axis. If the footprint is longitudinally complete, i.e. measurable from the base of the heel to the distal end of the impression left by the hallux, this length is called total length. The width corresponds to the largest breadth of the forefoot impression and is measured along the mediolateral axis which is perpendicular to the longitudinal axis. For longitudinally complete footprints, the width-to-length ratio was determined.

#### Morphometric comparisons

The dimensions and ratio of the Matalascañas footprints were compared with those of the other footprints attributed to Neandertals based on data from the literature: the footprints from Le Rozel^23^, two of the Theopetra footprints^24^, the complete Vârtop footprint^25^ and the Catalan Bay footprint^13^. The attribution of the latter footprint to a Neandertal can however be debated because of the lack of consensus on these dates combined with the fact that the footprint would correspond to the last Neandertal occurrence^26^.

#### Stature estimates

Statures were estimated from the total length of the footprints using the average of the estimates obtained from three relationships. The first relationship, commonly used in palaeobiological estimates from hominin footprints^27,28^, is based on a 15% ratio between foot length and stature. The other two relationships take into account the variations that may exist between footprint and the length of the associated foot. They are the result of experimental studies in which individuals walked barefoot in a substrate relatively close to that of Matalascañas. This is the relationship used by Dingwall and his collaborators^29^, on habitually unshod individuals, in the study of Ileret's footprints, and that of Duveau et al.^5^ on usually shod individuals, in the study of the footprints from Le Rozel. Methodological differences in the measurement of the total length of footprints exist between the relationships that we used. The total length of the Matalascañas footprints was measured from the heel to the tip of the hallux impresión; Dingwall et al.^29^ measured the distance from the base of the heel to the tip of the longest toe (the hallux or second toe); Duveau et al.^5^ considered the distance from the base of the heel to the second toe. However, anthropometric studies on foot and footprints have shown that the differences between the two types of lengths (heel base to first toe vs. heel base to second toe) are small, generally less than 1%^30–33^. We thus assume that such small differences cannot significantly affect estimates of stature and age class. For this reason, these estimates were made using regression equations independently of the methodology used to measure total length.

#### Age class estimates

An age class (children, adolescents, adults) was determined from the estimated statures for each footprint. The model established by Duveau et al.^5^ during the study of the footprints from Le Rozel was used for this purpose. This model represents the variation in stature as a function of age in Neandertals established from the osteological remains of 36 Neandertal individuals including 11 children and 25 adults (5 females, 14 males, 6 individuals of undetermined sex). For each longitudinally complete footprint, an age class was determined by placing the estimated statures on the curve of this model.

#### Minimum number of individual estimates (MNI)

An ichnological assemblages, like the HTS of Matalascañas footprints, spreading over a single surface represents a snapshot of life moments. The study of footprints can thus provide information on a number of individuals present on very short time scales^1,5^. HTS of Matalascañas are mostly considered isolated because it is often impossible to identify groups of footprints made by one individual (or “trackway”). Therefore, the counting of individuals who made these footprints is more complex. In order to determine a Minimum Number of Individuals (MNI), the intraindividual dispersion of footprint total length was used. This is based on the fact that footprints of a single individual can vary in length but that this variation is limited. By knowing the limits of this variation, it is then possible to determine a MNI. We thus used the intraindividual dispersion experimentally determined during the study of the Neandertal footprints from Le Rozel^5^. The use of these data is justified by the proximity in the depositional conditions of the footprints between Le Rozel and Matalascañas. According to the data of Duveau et al.^5^ the largest value of the maximum intraindividual deviations of the total length is equal to 12.8%. Therefore, we considered that Matalascañas footprint total lengths (L_tot_) falling within the interval [L_tot_ × (1 – 0.128); L_tot_ × (1 + 0.128)] from each other corresponded to footprints made by the same individual.

## Results

The level where the fossil tracks are recorded is just the upper surface of the palaeosol Ps2. This is a layer composed of medium and fine sands with a silty matrix (sample Ps-2, SI Appendix, Tables S1, S2, Figs. S1, S2). A matrix's presence gives this layer a cohesive character that allowed it to preserve the fossil tracks. Just on top of this tracked surface, a thin millimeter-scale bed of iron oxide is formed. Probably this lamina was developed from a synsedimentary microbial mat oxidized after deposition. This upper surface of Ps2 was preserved by the sands that constitute the base of U1. These are moderately sorted fine sands with a visible ripple-type cross lamination (Sample U-1, base). The rest of the unit U1 comprised bodies of cross-bedded poorly sorted medium sands (sample U1, 1.5 m) SI Appendix, Tables S1, S2, Figs. S1, S2) and were interpreted by Zazo et al.^22^ as aeolian migrating dunes. The rest of U2 and U3 are also constituted by cross-bedded sands with similar internal structure and interpretation. The units were separated using depositional criteria, taking into account non-depositional surfaces' presence accompanied by the development of edaphic horizons. In some segments of the column, not only the wind is the responsibility of the deposition. Some finer levels affected by fluid deformations (possibly liquefaction phenomena) are present between different cross-bedded bodies.

**Figure 4:**
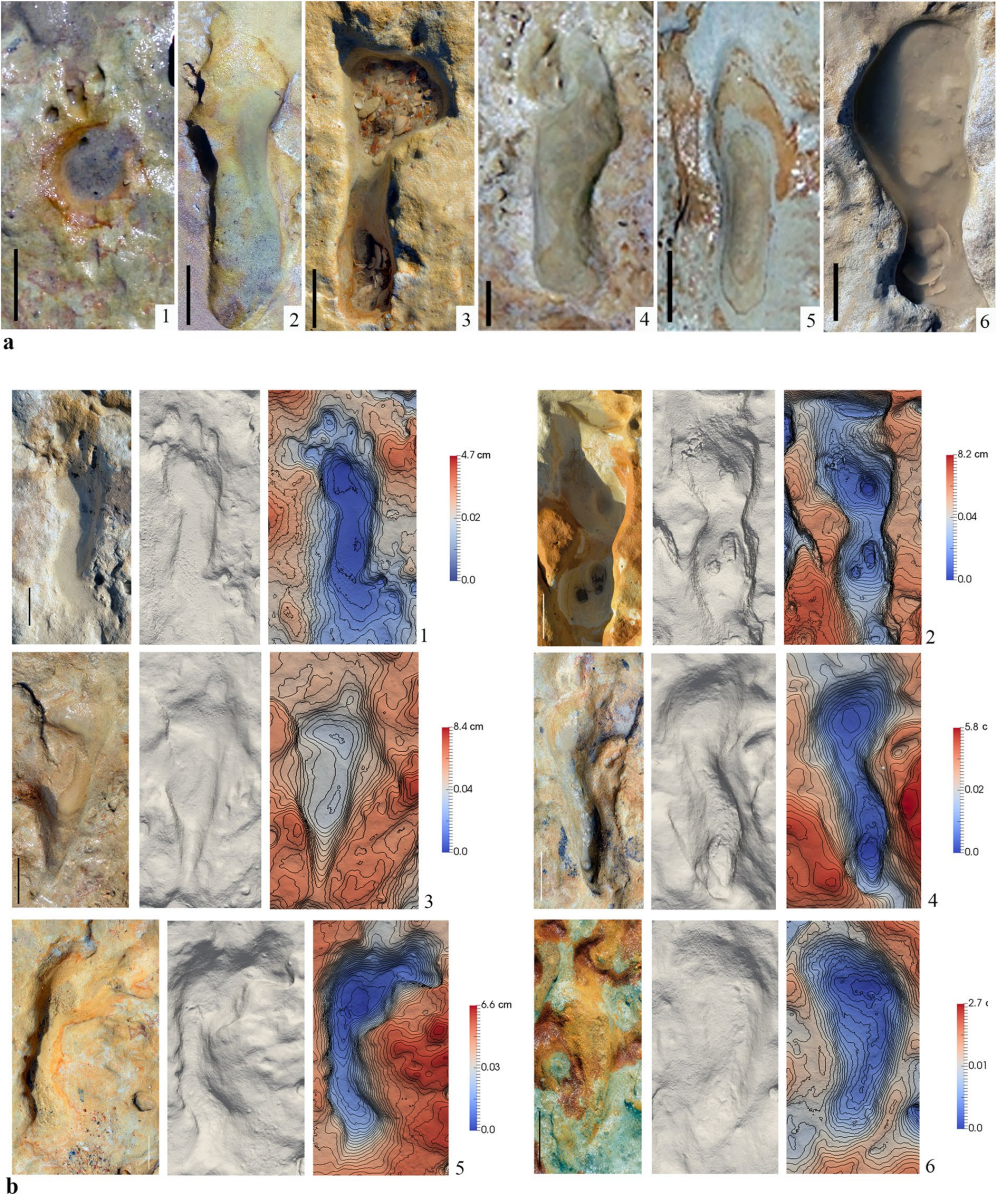
Hominin footprints from Matalascañas site. (**a**) Detail view of some footprints. (**a1**) M2020-93, (**a2**) M2020-17, (**a3**) M2020-15, (**a4**) M2020-06, (**a5**) M2020-08, (**a6**) M2020-14. Scale bar, 5 cm. Natural views, solid models and shaded 3D elevation with contour lines images. (**b1**) M2020-12, (**b2**) M2020-22, (**b3**) M2020-19, (**b4**) M2020-18, (**b5**) M2020-20, (**b6**) M2020-21. Scale bar, 5 cm.

All the hominin footprints are preserved as concave epirreliefs in a substrate soft enough, but consistent, to preserve quite well the morphology and contour of the feet (Fig. 4a,b). So far, 87 hominin footprints have been recognized at the Matalascañas site, 41 corresponds to left feet (Fig. 4a1,a3,a4,a6,b3,b4,b5, and SI Appendix, Table S4) and 46 to the right (Fig. 4a2,a6,b1,b2,b6 and SI Appendix, Table S4).

Until now, four pairs of consecutive footprints (SI Appendix, Table S5) were identify with pace angles ranging from 4° to 13°. The rest of the footprints are considered isolated. Several of the footprints, such as the M2020-12 (Fig. 4a1,b1), reflect a high level of anatomical details by showing, for example, clear toe impressions.

Thanks to the anatomical details that they reflect, the footprint assemblage from Matalascañas can be undoubtedly related to hominin. Indeed, they show several anatomical features such as a rounded heel, a longitudinal arch, relatively short toes, and adducted hallux^34,35^. The maximum depth positions of the footprints are located beneath the heel and the forefoot, which is consistent with the distribution of plantar pressures during human bipedal walking^36,37^. (Fig. 4b). In some cases, the deeper area does not correspond precisely to the heel or the forefoot (Fig. 4a8). This reason is due to the erosion produced by small stones or shells which, trapped in the footprint cavity, were carried away by the continuous action of the tides.

The length of the 87 measured footprints measured (Table 1a and SI Appendix Table S4) range from 14 to 29 cm (mean: 21 cm) and their width (w) from 4 to 13 cm (mean: 7 cm) (SI Appendix Table S5). Among these 87 footprints, 31 are longitudinally complete, from the base of the heel to the tip of the second toe impression, with total lengths (L_tot_) ranging from 14 to 29 cm (mean: 21 cm). The ratio between width and total length varies from 0.22 to 0.50 (average: 0.37). Total length and width are relatively uncorrelated (r^2^ = 0.5).

**Table 1:** Measurements of Matalascañas' footprints.

**a**	n	Min	Mean	Max	s.d								
Total length (cm)	31	14	21	29	4								
Width (cm)	87	4	7	13	2								
Width/total length	31	0.22	0.37	0.50	0.06								

**b**													
Sites	Total length (cm)				Width (cm)				Width/total length				References
	n	Min	Mean	Max	n	Min	Mean	Max	n	Min	Mean	Max	
Matalascañas	31	14	21	29	78	4	7	12	31	0.22	0.37	0.5	This paper
Le Rozel	54	11.4	19.2	28.4	126	4.5	8.4	12.8	49	0.32	0.43	0.56	Duveau^23^
Theopetra	2	13.8	14.4	15.2	2	5.4	5.8	6.3	2	0.36	0.41	0.45	Manolis et al.^24^
Vârtop	1		22		1		10.6		1		0.48		Onac et al.^25^
Catalan Bay	1		17		1		7		1		0.41		Muñiz et al.^13^

**c**	Estimated stature (cm)												
	n	Min	Mean	Max	s.d								
	31	104	144	188	25								

**d**	Age class												
		Children (< 10 years)	Adolescents (10–18 years)	Adults (> 18 years)									
	n	7	15	9									
	Relative frequency (%)	23	48	29									

(**a**) Range of values. (**b**) Size comparison with other sites. (**c**) Estimated stature. (**d**) Estimated hominin group composition.

**Figure 5:**
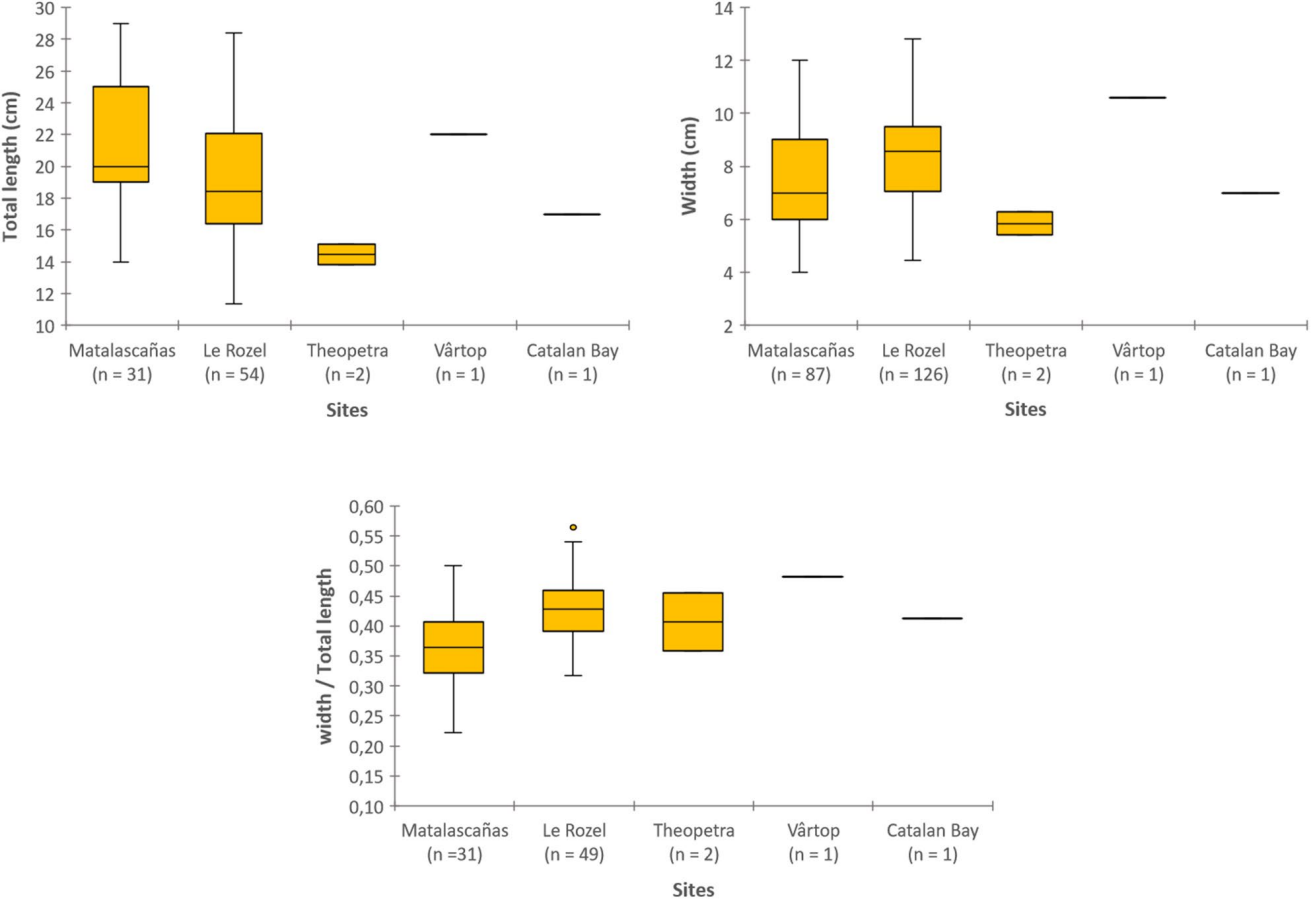
Comparisons of dimensions (total length and width) and proportions (width/total length) between the Matalascañas footprints and the other footprints attributed to Neandertals: the footprints from Le Rozel^5^, two of the Theopetra footprints^23^, the complete Vârtop footprint^24^, and the Catalan Bay footprint^13^.

The dispersion of the total lengths and widths of the Matalascañas footprints are large (Table 1b, Fig. 5 and SI Appendix Tables S6, S7) and are close to those from Le Rozel (L_tot_: 11.4–28.4 cm; w: 4.5–12.8 cm). They are on average longer (mean L_tot_: 21 cm) than the Theopetra footprints (mean: 14.4 cm) and that from Catalan bay (mean: 17 cm) but less than that the Vârtop footprint (mean: 22 cm). The ratio between the width and length of the Matalascañas footprints is highly dispersed, this morphological variability being common for footprints made in soft sediments, but is lower on average than for the other Neandertal footprints (mean: 0.43) (Fig. 5, SI Appendix Table S8).

**Figure 6:**
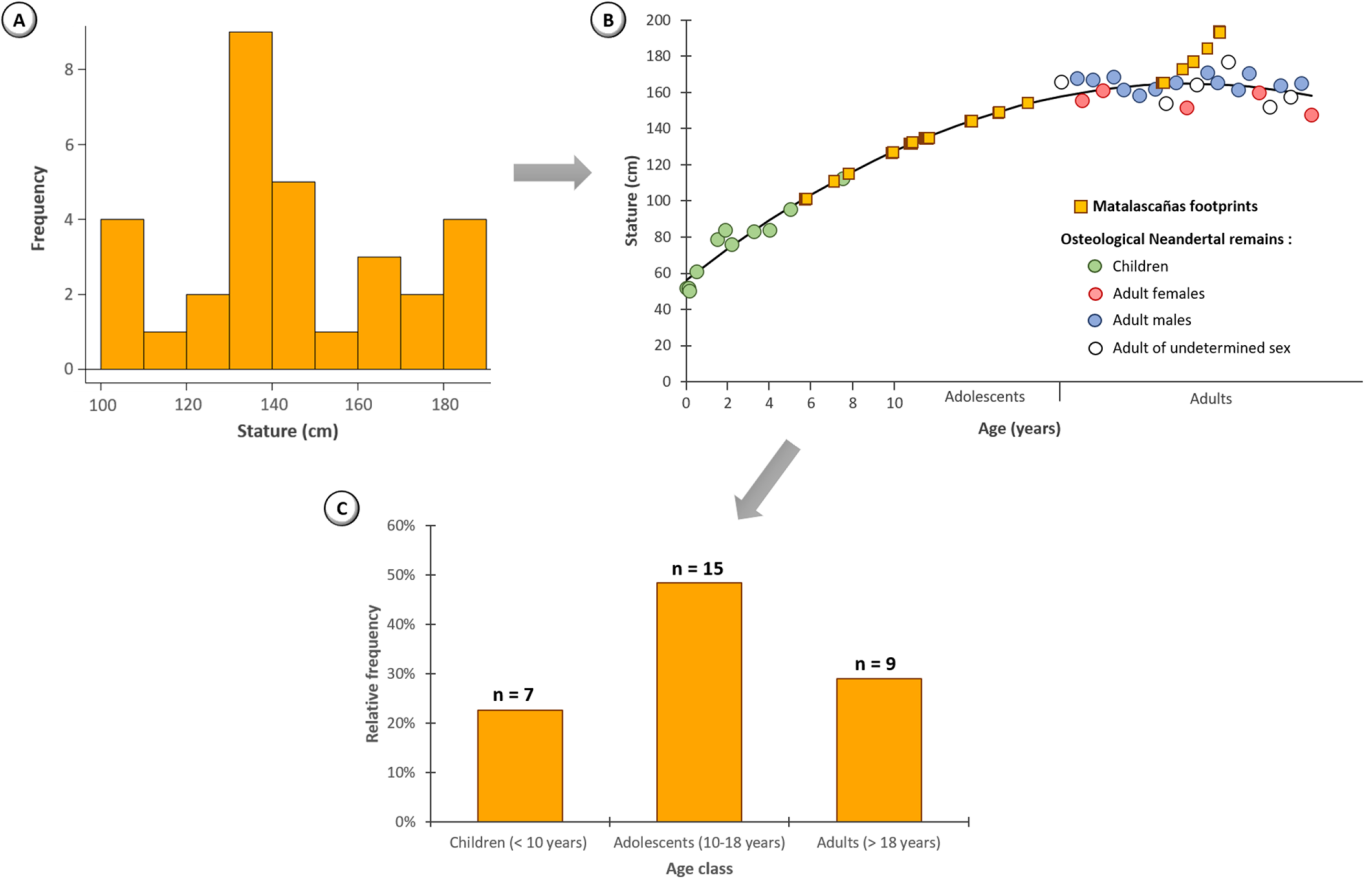
Palaeobiological estimates based on the 31 longitudinally complete footprints from Matalascañas. (**a**) Distribution of estimated statues. (**b**) Position of the estimated statures on a curve representing the stature variation with age for Neandertals defined from osteological remains^5^. (**c**) Distribution of estimated age classes.

The statures estimated from the lengths of the 31 longitudinally complete footprints range from 104 to 188 cm (mean: 144 cm) (Table 1c, Fig. 6a). The statures are described by a normal distribution (Shapiro–Wilk test: P = 0.13). Almost half of the footprints correspond to statures between 130 and 150 cm.

The 31 estimated statures correspond to different age groups (Table 1d; Fig. 6b,c, and SI Appendix Table S9): from children to adults. Overall, 7 footprints are associated with children, 15 with adolescents and 9 with adults. The 2 smallest (L_tot_ = 14 cm) footprints correspond to an age of approximately 6 years. 11 footprints are at the limit between children and adolescents. Therefore, the association to an age class for these footprints is not completely certain. Moreover, 5 footprints correspond to statures between 140 and 155 cm and were associated with adolescents according to the model. However, they could also have been made by adult Neandertal females or small males. Finally, the 4 longest footprints (L_tot_ = 28–29 cm) correspond to statures (182–188 cm) which are larger than the largest estimated stature on a Neandertal skeleton (Duveau et al.^5^ site Amud 1, Israel with 177 cm).

A count of individuals from the reported consecutive footprints cannot be carried out since only one footprint is well enough preserved to have access to the total length. However, a Minimum Number of Individuals can be estimated from isolated footprints based on experimental knowledge of the intraindividual dispersion of the footprint total lengths. The 31 longitudinally complete footprints thus correspond to at least 3 individuals. They were left by at least one child with a stature between 104 and 115 cm for an age ranging from 6 to 8 years, one individual with a stature between 126 and 149 cm which corresponds to an adolescent or small adult, and one tall adult (154–188 cm) who was probably a man given the sexual dimorphism present in the Neanderthals^38,39^.

## Discussion

Neandertals seem to be the most likely track-makers from the chronological and the archaeological context on the HTS of Matalascañas. Indeed, on the one hand, the age established for the Pleistocene formation including the Matalascañas footprints (during the upper Pleistocene before 106 ± 19 kyr) coincides with the time when the Neandertals were the only hominin species known in Western Europe^40,41^.

Similar to most of the sites where hominin footprints have been found, no palaeoanthropological skeletal remains have been discovered at Matalascañas associated with the footprints. Nevertheless, recently appeared several pieces of lithic industry on the surface of the outcrop, that is still under investigation. The presence of this industry indicates a Mousterian age (García Rincón, pers. comm. 2020) which would also confirm this taxonomic attribution^42^. In fact, in the surroundings of Matalascañas were located in 1985, several pieces and remains of carvings belonging to the Middle Paleolithic, which reinforces the previous hypothesis^43^. The association of the lithic industry with hominin footprints is an infrequent feature and is only known through a few occurrences around the world^5,7^.

The footprints of the HTS of Matalascañas site are the oldest in the world for Neandertals during the Upper Pleistocene, somewhat older than the three isolated footprints from the site of Vârtop (Romania), with an age of 97–62 kyr^25,44,45^, than the 257 footprints in Le Rozel France, 80 kyr^5^ and much older than the potential footprint from Gibraltar, 28 kyr^13^.

### Palaeoenvironmental reconstruction

**Figure 7:**
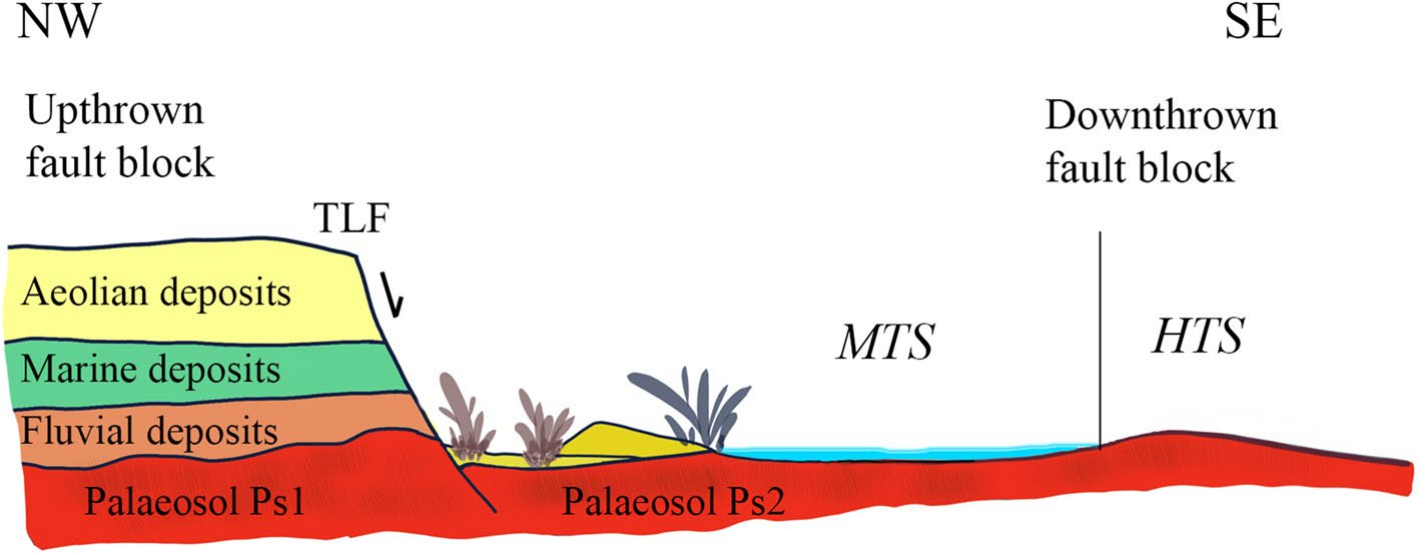
Section showing the stratigraphic order of units exposed in the El Asperillo cliff during the Last Interglacial (OIS5)^22^. Note the wide extension of the palaeosol surface where all hominid (HTS) and other vertebrates (MTS) footprints were recorded. TFL: Torre del Loro Fault.

According to previous research^22,46,47^, the area where the outcrop is located would correspond to a downthrown block generated by a fault (Torre del Loro Fault, TLF) that generated a faulted sea-cliff (an Asperillo palaeocliff), whose base would be constituted by the palaeosol (Ps2, Figs. 2c, 7). On this palaeosol, which formed a wide platform of almost 15 km in a NW–SE direction, an extensive flooded corridor developed covered by a thin layer of water that favoured the appearance of an extensive microbial mat. The presence and persistence of these benthic microbial mats are a common characteristic of hypersaline systems, such as lagoons and evaporative lakes^48^ whose environmental conditions are very similar to those of MTS where it is common to find external halite moulds on the surface of these mats (Fig. 2e). After prolonged subaerial exposure wide shrinkage cracks were developed with microbial growth probably induced by rising groundwater due to hydraulic upward pressure^49^ in a similar way as it happens today in the intertidal zone of El Jellabia; southern Tunisia^50^. Same polygonal mat with upturned crack margins to those of Matalascañas can also be observed nowadays in the tidal flat and coastal sabkha of southern Tunisia Bhar Alouane (Eriksson et al.^51^).

These swamped areas would probably be located very close to the coastline, even in an intertidal context very similar to the present-day one and they would be protected laterally by incipient dune chains. These dunes were accumulated against the fault scarp, giving rise to the U1 aeolian unit that would over time fossilize the surface of the palaeosol. It is in this context where the tracks of the large vertebrates (Artiodactyla, Elephantidae, Canidae, etc.) were produced, mainly in the flooded areas and of the hominin, in the areas bordering the previous ones (Fig. 3a,c,d).

### Behavioral considerations

An important aspect of these data is the possibility of being able to determine the behaviour of the individuals who are part of the group. Neandertals are hunter-gatherers^52^ so the reasons for their presence are mainly due to travel, transportation of resources or foraging strategies.

The group's estimated composition is dominated by non-adult individuals (71%), of which almost 25% are children. As shown in Fig. 3, most of the footprints (about 75%) are located outside the flooded area where the passage of large mammals and birds is located. Considering that the dominant direction of the footprints is SW-NE (Fig. 3b) in the sense of advancing or retiring towards the pools, several interesting hypotheses open up. First of all, most of the footprints are located on the edge of the flooded area, without moving entirely into it. This could involve a hunting strategy, stalking animals in the water, probably not the large artiodactyls or proboscidean, but waterbirds and waders or small carnivores or even fishing or shellfish search of fish or molluscs; The exploitation of similar preys by Neandertals for subsidence strategies and cultural behaviors has already been reported^53^. The hypothesis of the travel is not discarded either. However, the possibility of a resource transport is difficult to identify from footprint morphology, as experimental analyses have shown a small variation in footprint total length (less than one cm on average) when individuals are loaded or unloaded^54^. Nevertheless, these footprints' dominant direction is not NW–SE (Fig. 3b), as would be expected if the group were to border the waterlogged areas and move parallel to the coast.

Therefore, it should be noted that all the evidence seems to indicate that some activity was taking place around the pond and that the tracks do not correspond to a migration or passage path.

Although there is abundant archaeological material of Neandertals recorded in or near coastal areas^55–57^, such as stone industry, bone remains or consumption of marine vertebrates or molluscs, the presence of their footprints in this kind of environments is very scarce^5^. In the Iberian Peninsula different type of archaeological evidence has been described on the coast of Arrábida, Portugal^52^, Bajondillo and Abrigo 3 caves, in Malaga, Spain^58^ and Vanguard and Gorham caves in Gibraltar^59^, as well as the presence of a poorly preserved footprint in Gibraltar^13^. Nevertheless, the HTS of Matalascañas site is the first unequivocal record of Neanderthal tracks in Iberian coastal areas, which also has an associated lithic industry. So, this finding reinforces the ecological scenario in which Neanderthal groups made extensive use, at least temporarily, of coastal environments.

## Conclusions

The hominin footprints studied herein were impressed at the top of a fine to medium sandstone that belongs to a palaeosol (Ps2) and is covered by the base of an aeolian unit (U1) of about 106 ± 19 kyr. The presence of benthic microbial mats, as well as its sedimentological features, suggests a coastal environment, more specifically, a shoreline of a hypersaline swamped area under an intertidal context. Morphological characteristics undoubtedly relate these tracks to hominins, and due to their geological age, they belong to Neandertals. They complete a very fragmented Neandertal ichnological record and represent the oldest Neandertal footprints in the world during Upper Pleistocene. According to the morphometric comparisons made with the complete footprints, the statures of the producers vary from 104 to 188 cm (mean: 144 cm), being 7 footprints are associated with children, 15 with adolescents and 9 with adults. The 2 smallest footprints, of 14 cm, corresponding to an age of approximately 6 years. The 4 longest footprints (28–29 cm) correspond to statures (182–188 cm), which are larger than the largest estimated stature on a Neandertal skeleton, although it must be kept in mind that these prints could have been left by a smaller individual because of the intraindividual morphometric dispersion of footprints. Thanks to the experimentally knowledge of this dispersion a Minimum Number of 3 Individuals was estimated. The distribution of Neandertal tracks are outside the flooded area where the passage of large mammals and birds is located, and their orientation is mainly perpendicular to the shoreline. The data obtained in this work suggest that the studied footprints were impressed by a multi-aged group of Neandertals dominated by non-adults, and support the ecological relationships between Neandertal populations and coastal areas.

## Supplementary Information

Supplementary Information.

## References

[1] Bennett MR, Morse SA (2014). Human Footprints: Fossilised Locomotion?.

[2] Leakey MD, Hay RL (1979). Pliocene footprints in the Laetolil Beds at Laetoli, northern Tanzania. Nature.

[3] Mietto P, Avanzini M, Rolandi G (2003). Palaeontology: Human footprints in Pleistocene volcanic ash. Nature.

[4] Ashton N (2014). Hominin footprints from early Pleistocene deposits at Happisburgh, UK. PLoS ONE.

[5] Duveau J (2019). The composition of a Neandertal social group revealed by the hominin footprints at Le Rozel (Normandy, France). PNAS.

[6] Masao FT (2016). New footprints from Laetoli (Tanzania) provide evidence for marked body size variation in early hominins. eLife.

[7] Altamura F (2018). Archaeology and ichnology at Gombore II-2, Melka Kunture, Ethiopia: Everyday life of a mixed-age hominin group 700,000 years ago. Sci. Rep..

[8] Bustos D (2018). Footprints preserve terminal Pleistocene hunt? Human-sloth interactions in North America. Sci. Adv..

[9] Stewart M (2020). Human footprints provide snapshot of last interglacial ecology in the Arabian interior. Sci. Adv..

[10] Barton CM (1990). Stone tools and paleolithic settlement in the Iberian Peninsula. Proc. Prehist. Soc..

[11] Garralda MD (2005). The Neandertals from the Iberian Peninsula. MUNIBE.

[12] Ruiz MN (2020). Last Neandertal occupations at Central Iberia: The lithic industry of Jarama VI rock shelter (Valdesotos, Guadalajara, Spain). Archaeol. Anthropol. Sci..

[13] Muñiz F (2019). Following the last Neandertals: Mammal tracks in Late Pleistocene coastal dunes of Gibraltar (S Iberian Peninsula). Quat. Sci. Rev..

[14] Neto de Carvalho C (2020). First vertebrate tracks and palaeoenvironment in a MIS-5 context in the Doñana National Park (Huelva, SW Spain). Quat. Sci. Rev..

[15] Neto de Carvalho C (2020). Paleoecological implications of large-sized wild boar tracks recorded during the last interglacial (Mis 5) at Huelva (Sw Spain). Palaios.

[16] Rodríguez-Ramírez A (2014). The role of neo-tectonics in the sedimentary infilling and geomorphological evolution of the Guadalquivir estuary (Gulf of Cadiz, SW Spain) during the Holocene. Geomorphology.

[17] Rodríguez-Rámirez, A. Geomorfología del Parque Nacional de Doñana y su Entorno. (ed Organismo Autónomo Parques Nacionales) (Ministerio de Medio Ambiente, Madrid, 1998).

[18] Pérez Muñoz, A. B. *et al*. Parque Nacional de Doñana. Guía Geológica. (ed Rodríguez Fernández, R.) (Instituto Geológico y Minero de España & Organismo Autónomo Parques Nacionales, Madrid, 2020).

[19] Instituto Hidrográfico de la Marina (1992). Derrotero N° 2-Tomo 2.

[20] Rodríguez-Ramírez A, Ruiz A, Cáceres L (2003). Analysis of the recent storm record in the southwestern spanish coast: Implications for littoral management. Sci. Total Environ..

[21] Gibbard PL, Head MJ, Walker MJC, The Subcommission on Quaternary Stratigraphy (2010). Formal ratification of the Quaternary System/Period and the Pleistocene Series/Epoch with a base at 2.58 Ma. J. Quat. Sci..

[22] Zazo C (2005). Landscape evolution and geodynamic controls in the Gulf of Cadiz (Huelva coast, SW Spain) during the Late Quaternary. Geomorphology.

[23] Duveau, J. *Les empreintes de pieds du Rozel (Manche). Instantanés de groupes humains au Pléistocène supérieur. Approche combinée morphométrique et expérimentale*. (Ph. D. dissertation. Muséum national d’Histoire naturelle, Paris, 2020).

[24] Manolis, S., Aiello, L., Henessy, R., Kyparissi-Apostolika, N. Middle Palaeolithic Footprints from Theopetra Cave (Thessaly, Greece) (ed Kyparissi-Apostolika, N.) 87–93 (Greek Ministry of Culture and Institute for Aegean Prehistory, Athens, 2000).

[25] Onac BP, Viehmann I, Lundberg J, Lauritzen S-E (2005). U-Th ages constraining the Neanderthal footprint at Vârtop Cave, Romania. Quat. Sci. Rev..

[26] Duveau, J., Berillon, G., Verna, C. 11-On the tracks of Neandertals: The ichnological assemblage from Le Rozel (Normandy, France). (eds Pastoors, A. & Lenssen-Erz, T.) (Springer Nature, in Press).

[27] Citton P, Romano M, Salvador I, Avanzini M (2017). Reviewing the upper Pleistocene human footprints from the ‘Sala dei Misteri’in the Grotta della Basura (Toirano, northern Italy) cave: An integrated morphometric and morpho-classificatory approach. Quat. Sci. Rev..

[28] Helm CW, McCrea RT, Cawthra HC (2018). A New Pleistocene Hominin Tracksite from the Cape South Coast, South Africa. Sci. Rep..

[29] Dingwall HL, Hatala KG, Wunderlich RE, Richmond BG (2013). Hominin stature, body mass, and walking speed estimates based on 1.5 million-year-old fossil footprints at Ileret, Kenya. J. Hum. Evol..

[30] Krishan K (2008). Estimation of stature from footprint and foot outline dimensions in Gujjars of North India. Forensic Sci. Int..

[31] Fawzy IA, Kamal NN (2010). Stature and body weight estimation from various footprint measurements among Egyptian population. J. Forensic Sci..

[32] Reel S, Rouse S, Obe WV, Doherty P (2012). Estimation of stature from static and dynamic footprints. Forensic Sci. Int..

[33] Hemy N, Flavel A, Ishak NI, Franklin D (2013). Sex estimation using anthropometry of feet and footprints in a Western Australian population. Forensic Sci. Int..

[34] Aiello L, Dean C (1990). An Introduction to Human Evolutionary Anatomy.

[35] Klenerman L, Wood B (2006). The Human Foot: A Companion to Clinical Studies.

[36] Elftman H, Manter J (1935). Chimpanzee and human feet in bipedal walking. Am. J. Phys. Anthropol..

[37] Alexander RM (2003). Principles of Animal Locomotion.

[38] Ruff CB, Trinkaus E, Holliday TW (1997). Body mass and encephalization in Pleistocene Homo. Nature.

[39] Carretero JM (2012). Stature estimation from complete long bones in the Middle Pleistocene humans from the Sima de los Huesos, Sierra de Atapuerca (Spain). J. Hum. Evol..

[40] Benazzi S (2011). Early dispersal of modern humans in Europe and implications for Neandertal behaviour. Nature.

[41] Hublin JJ (2015). The modern human colonization of western Eurasia: When and where?. Quat. Sci. Rev..

[42] Karavanić I (2018). Paleolithic hominins and settlement in Croatia from MIS 6 to MIS 3: Research history and current interpretations. Quat. Int..

[43] Vallespi, E., Alvarez, G., Perez Sindreu, F. & Rufete, P. Nuevas atribuciones onubenses al Paleolitico Inferior y Medio. Huelva en su Historia I, 43–56 (1986).

[44] Viehmann I (1987). Prehistoric Human Footprints in Romania’s Caves. Theor. Appl. Karstol..

[45] Harvati, K. The human fossil record from Romania: Early Upper Paleolithic European Mandibles and Neanderthal. (eds Harvati, K. & Roksandic, M.) 51–68 (Springer Netherlands, 2016).

[46] Zazo C (1999). Pleistocene and Holocene Aeolian facies along the Huelva coast (southern Spain): Climatic and neotectonic implications. Geol. Mijn..

[47] Zazo, C. *et al*. El complejo eólico de El Abalario (Huelva) (eds Sanjaume, E., Gracia, F. J.) 407–425 (Sociedad Española de Geomorfología, Madrid, 2011)

[48] Paerl, H. W. & Yanarell, A. C. Environmental dynamics, community structure and function in a hypersaline microbial mat (eds Seckbach, J. & Oren, A.) 421–442, (Springer Netherlands, 2010).

[49] Porada, H. & Bouougri, E. Wrinkle structures—a critical review (eds Schieber, J. et al.) 135–144 (Elsevier, 2007).

[50] Gerdes, G. What Are Microbial Mats? (eds Seckbach, J. & Oren, A.) 3–25, (Springer Netherlands, 2010).

[51] Eriksson, P. G. *et al*. Paleoenvironmental Context Of Microbial Mat-Related Structures In Siliciclastic Rocks. (eds Seckbach, J. & Oren, A.) 71–108 (Springer Netherlands, 2010).

[52] Zilhão J (2020). Last Interglacial Iberian Neandertals as fisher-hunter-gatherers. Science.

[53] Hardy BL, Moncel M-H (2011). Neanderthal use of fish, mammals, birds, starchy plants and wood 125–250,000 years ago. PLoS ONE.

[54] Wall-Scheffler CM, Wagnild J, Wagler E (2015). Human footprint variation while performing load bearing tasks. PLoS ONE.

[55] Romagnoli F, Martini F, Sarti L (2015). Neanderthal use of *Callista chione* shells as raw material for retouched tools in South-East Italy: Analysis of Grotta del Cavallo layer l assemblage with a new methodology. J. Archaeol. Method Theory.

[56] Benito BM (2017). The ecological niche and distribution of Neanderthals during the Last Interglacial. J. Biogeogr..

[57] Villa P (2020). Neandertals on the beach: Use of marine resources at Grotta dei Moscerini (Latium, Italy). PLoS ONE.

[58] Cortés-Sánchez M (2019). Shellfish collection on the westernmost Mediterranean, Bajondillo cave (~ 160–35 cal kyr BP): A case of behavioral convergence?. Quat. Sci. Rev..

[59] Stringer CB (2008). Neandertal exploitation of marine mammals in Gibraltar. PNAS.

