# Supplementary material for: Tracking late Pleistocene Neandertals on the Iberian coast
Source: Sci Rep. 2021 Mar 11;11:4103. doi: 10.1038/s41598-021-83413-8 (PMC7952904; doi:10.1038/s41598-021-83413-8)
Supplement: Supplementary file 1 — Supplementary Information. [file 41598_2021_83413_MOESM1_ESM.docx]

**Supplementary Information:**

Tracking late Pleistocene Neandertals on the Iberian coast

E. Mayoral^1,2*^, I. Díaz-Martínez^3,4^, J. Duveau^5^, A. Santos^2^, A. Rodríguez Ramírez^1,2^, J.A. Morales^1,2,^ L.A. Morales^6^ & R. Díaz-Delgado^7^

^1^Departamento de Ciencias de la Tierra, Facultad de Ciencias Experimentales, Campus de el Carmen, Universidad de Huelva, Huelva, Spain

^2^CCTH - Centro de Investigación Científico Tecnológico, Universidad de Huelva, Huelva, Spain

^3^Instituto de Investigación en Paleobiología y Geología (IIPG). CONICET. General Roca. Río Negro, Argentina

^4^Universidad Nacional de Río Negro-IIPG. General Roca. Río Negro. Argentina

^5^Histoire Naturelle de l’Homme Préhistorique, CNRS, Muséum National d’Histoire Naturelle, Université Perpignan Via Domitia, Paris, France

^6^ Al Futuro Arquitectura, Huelva, Spain

^7^ Estación Biológica de Doñana-CSIC, Sevilla, Spain

*Email: [mayoral@dgeo.uhu.es](mailto:mayoral@dgeo.uhu.es)

**Table S1.** Sediment grain-size results for samples collected in some units of the sedimentary column. Results expressed in percentage by weight. VCS: Very Coarse Sand, CS: Coarse Sand, MS: Medium Sand, FS: Fine Sand, VFS: Very Fine Sand. UPL: Upper Plastic Level.

|  | | **Average size**  **(ø units)** | **Average size**  **(mm)** | **PS-2** | **U-1 (BASE)** | **U-1 (1,5m)** | **U-2 (UPL)** |
| --- | --- | --- | --- | --- | --- | --- | --- |
| **GRAVEL** | | **-2.5** | **3.000** | 0.00 | 0.00 | 0.00 | 0.00 |
| **SAND** | **VCS** | **-0.5** | **1.500** | 0.00 | 2.36 | 2.81 | 0.13 |
|  | **CS** | **0.5** | **0.750** | 0.69 | 15.30 | 19.34 | 0.33 |
|  | **MS** | **1.5** | **0.375** | 42.66 | 25.71 | 41.80 | 9.85 |
|  | **FS** | **2.5** | **0.188** | 38.19 | 49.18 | 29.88 | 50.85 |
|  | **VFS** | **3.5** | **0.094** | 14.66 | 5.46 | 4.89 | 23.96 |
| **SILT** | | **4.5** | **0.032** | 3.79 | 2.00 | 1.28 | 14.89 |

**Table S2.** Accumulated sediment grain-size results for the analyzed samples. Results expressed in percentage by weight. VCS: Very Coarse Sand, CS: Coarse Sand, MS: Medium Sand, FS: Fine Sand, VFS: Very Fine Sand. UPL: Upper Plastic Level.

|  | | **Average size**  **(ø units)** | **Average size**  **(mm)** | **PS-2** | **U-1 (BASE)** | **U-1**  **(to 1.5m)** | **U-2 (UPL)** |
| --- | --- | --- | --- | --- | --- | --- | --- |
| **GRAVEL** | | **-2.5** | **3.000** | 0.00 | 0.00 | 0.00 | 0.00 |
| **SAND** | **VCS** | **-0.5** | **1.500** | 0.00 | 2.36 | 2.81 | 0.13 |
|  | **CS** | **0.5** | **0.750** | 0.69 | 17.65 | 22.15 | 0.46 |
|  | **MS** | **1.5** | **0.375** | 43.36 | 43.37 | 63.96 | 10.30 |
|  | **FS** | **2.5** | **0.188** | 81.54 | 92.55 | 93.83 | 61.15 |
|  | **VFS** | **3.5** | **0.094** | 96.21 | 98.00 | 98.72 | 8511 |
| **SILT** | | **4.5** | **0.032** | 100.00 | 100.00 | 100.00 | 100.00 |


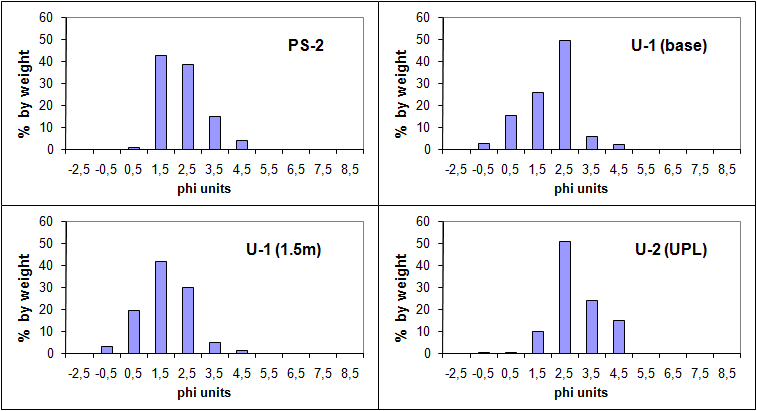
**Figure S1.** Histograms representing the sediment grain-size distribution for the analyzed samples.


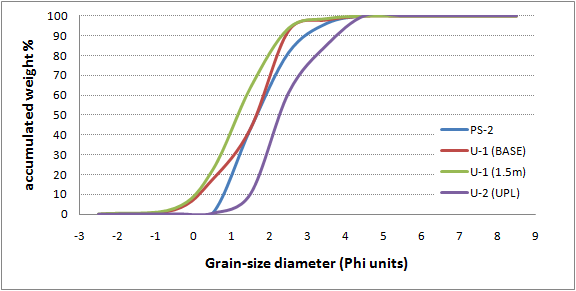


**Figure S2.** Curves representing the accumulated sediment grain-size distribution for the analyzed samples.

**Table S3.** Summary of the grain-size fractions for the analyzed samples. Results expressed in percentage by weight.

| **SAMPLE** | **G7S-1** | **G7S-2** | **G7S-3** | **G7S-4** |
| --- | --- | --- | --- | --- |
| **% gravel** | 0.00 | 0.00 | 0.00 | 0.00 |
| **% sand** | 96.21 | 98.00 | 98.72 | 85.11 |
| **% silt** | 3.79 | 2.00 | 1.28 | 14.89 |
| **% clay** | 3.79 | 2.00 | 1.28 | 14.89 |

**Table S4.** Inventory of the isolated footprints discovered at Matalascañas site.

| **Inventory number** | **Laterality** | **Length (cm)** | **Total length (cm)** | | **Width (cm)** | **Width / Total Length** |
| --- | --- | --- | --- | --- | --- | --- |
| M2020-01 | right | 20 | 20 | | 6 | 0.30 |
| M2020-02 | right | 19 | 19 | | 9 | 0.47 |
| M2020-03 | left | 18 | 18 | | 4 | 0.22 |
| M2020-04 | right | 13 | unknown | | 5 | unknown |
| M2020-05 | left | 18 | unknown | | 5 | unknown |
| M2020-06 | left | 29 | 29 | | 10 | 0.34 |
| M2020-07 | left | 15 | 15 | | 6 | 0.40 |
| M2020-08 | right | 21 | 21 | | 6 | 0.29 |
| M2020-09 | right | 19 | 19 | | 7 | 0.37 |
| M2020-10 | right | 14 | 14 | | 6 | 0.43 |
| M2020-11 | left | 25 | 25 | | 9 | 0.36 |
| M2020-12 | right | 22 | 22 | | 9 | 0.41 |
| M2020-13 | right | 23 | unknown | | 9 | unknown |
| M2020-14 | left | 27 | 27 | | 11 | 0.41 |
| M2020-15 | left | 28 | 28 | | 11 | 0.39 |
| M2020-16 | right | 28 | 28 | | 9 | 0.32 |
| M2020-17 | right | 29 | 29 | | 8 | 0.28 |
| M2020-18 | left | 23 | 23 | | 8 | 0.35 |
| M2020-19 | right | 18 | 18 | | 8 | 0.44 |
| M2020-20 | left | 26 | 26 | | 12 | 0.46 |
| M2020-21 | right | 16 | 16 | | 5 | 0.31 |
| M2020-22 | right | 21 | 21 | | 7 | 0.33 |
| M2020-23 | right | 25 | 25 | | 9 | 0.36 |
| M2020-26 | left | 21 | unknown | | 6 | unknown |
| M2020-27 | left | 25 | 25 | | 8 | 0.32 |
| M2020-28 | left | 22 | unknown | | 6 | unknown |
| M2020-29 | right | 14 | 14 | | 5 | 0.36 |
| M2020-30 | right | 24 | unknown | | 10 | unknown |
| M2020-31 | right | 14 | 14 | | 7 | 0.50 |
| M2020-32 | right | 15 | unknown | | 4 | unknown |
| M2020-33 | right | 27 | unknown | | 9 | unknown |
| M2020-34 | right | 18 | unknown | | 5 | unknown |
| M2020-36 | left | 15 | unknown | | 6 | unknown |
| M2020-37 | left | 15 | unknown | | 8 | unknown |
| M2020-38 | right | 28 | unknown | | 11 | unknown |
| M2020-40 | left | 20 | 20 | | 8 | 0.40 |
| M2020-42 | left | 21 | unknown | | 8 | unknown |
| M2020-43 | right | 20 | unknown | | 5 | unknown |
| M2020-45 | right | 15 | unknown | | 4 | unknown |
| M2020-48 | left | 13 | unknown | | 4 | unknown |
| M2020-49 | left | 28 | unknown | | 11 | unknown |
| M2020-51 | right | 19 | unknown | | 6 | unknown |
| M2020-52 | left | 21 | unknown | | 8 | unknown |
| M2020-55 | right | 22 | unknown | | 8 | unknown |
| M2020-56 | left | 25 | unknown | | 8 | unknown |
| M2020-57 | right | 19 | unknown | | 9 | unknown |
| M2020-58 | right | 20 | 20 | | 8 | 0.40 |
| M2020-59 | right | 14 | unknown | | 6 | unknown |
| M2020-60 | left | 13 | unknown | | 4 | unknown |
| M2020-61 | left | 13 | unknown | | 5 | unknown |
| M2020-62 | right | 14 | unknown | | 5 | unknown |
| M2020-63 | left | 15 | unknown | | 7 | unknown |
| M2020-64 | right | 14 | unknown | | 6 | unknown |
| M2020-65 | right | 16 | unknown | | 6 | unknown |
| M2020-66 | right | 16 | unknown | | 6 | unknown |
| M2020-67 | right | 28 | unknown | | 10 | unknown |
| M2020-68 | right | 22 | 22 | | 8 | 0.36 |
| M2020-69 | left | 21 | unknown | | 7 | unknown |
| M2020-71 | left | 15 | unknown | | 5 | unknown |
| M2020-72 | left | 15 | unknown | | 5 | unknown |
| M2020-73 | left | 26 | unknown | | 10 | unknown |
| M2020-74 | left | 18 | unknown | | 9 | unknown |
| M2020-76 | right | 24 | unknown | | 8 | unknown |
| M2020-77 | right | 15 | unknown | | 6 | unknown |
| M2020-78 | right | 23 | unknown | | 9 | unknown |
| M2020-79 | left | 24 | unknown | | 11 | unknown |
| M2020-80 | left | 16 | unknown | | 6 | unknown |
| M2020-81 | left | 19 | unknown | | 8 | unknown |
| M2020-82 | left | 19 | unknown | | 6 | unknown |
| M2020-83 | right | 22 | unknown | | 6 | unknown |
| M2020-84 | right | 19 | 19 | | 7 | 0.37 |
| M2020-85 | right | 20 | 20 | | 8 | 0.40 |
| M2020-86 | left | 14 | unknown | | 5 | unknown |
| M2020-88 | left | 18 | unknown | | 9 | unknown |
| M2020-89 | left | 21 | 21 | | 7 | 0.33 |
| M2020-90 | left | 17 | unknown | | 7 | unknown |
| M2020-91 | left | 19 | unknown | | 8 | unknown |
| M2020-92 | right | 20 | 20 | | 9 | 0.45 |
| M2020-93 | left | 12 | unknown | 7 | | unknown |

**Table S5. Inventory of the footprints included in consecutive footprints discovered at Matalascañas site.**

| **Consecutive pair Inventory Number** | **Inventory Number** | **Laterality** | **Length**  **(cm)** | **Total length**  **(cm)** | **Width**  **(cm)** | **Width / Total Length** |
| --- | --- | --- | --- | --- | --- | --- |
| M2020-24-25 | M2020-24 | left | 21 | unknown | 8 | unknown |
|  | M2020-25 | right | 25 | unknown | 10 | unknown |
| M2020-46-47 | M2020-46 | right | 19 | unknown | 7 | unknown |
|  | M2020-47 | left | 19 | 19 | 5 | 0.26 |
| M2020-53-54 | M2020-53 | left | 19 | unknown | 6 | unknown |
|  | M2020-54 | right | 21 | unknown | 6 | unknown |
| M2020-41-44 | M2020-41 | right | 26 | unknown | 8 | unknown |
|  | M2020-44 | right | 23 | unknown | 9 | unknown |

**Table S6. Comparison of the total length of the footprints of Matalascañas site with the Theopetra. Vârtop and Catalán Bay sites.**

| **Site** | **Inventory number** | **Total Length (cm)** | **References** |
| --- | --- | --- | --- |
| Matalascañas | M2020-01 | 20 | This article |
| Matalascañas | M2020-02 | 19 |  |
| Matalascañas | M2020-03 | 18 |  |
| Matalascañas | M2020-06 | 29 |  |
| Matalascañas | M2020-07 | 15 |  |
| Matalascañas | M2020-08 | 21 |  |
| Matalascañas | M2020-09 | 19 |  |
| Matalascañas | M2020-10 | 14 |  |
| Matalascañas | M2020-11 | 25 |  |
| Matalascañas | M2020-12 | 22 |  |
| Matalascañas | M2020-14 | 27 |  |
| Matalascañas | M2020-15 | 28 |  |
| Matalascañas | M2020-16 | 28 |  |
| Matalascañas | M2020-17 | 29 |  |
| Matalascañas | M2020-18 | 23 |  |
| Matalascañas | M2020-19 | 18 |  |
| Matalascañas | M2020-20 | 26 |  |
| Matalascañas | M2020-21 | 16 |  |
| Matalascañas | M2020-22 | 21 |  |
| Matalascañas | M2020-23 | 25 |  |
| Matalascañas | M2020-27 | 25 |  |
| Matalascañas | M2020-29 | 14 |  |
| Matalascañas | M2020-31 | 14 |  |
| Matalascañas | M2020-40 | 20 |  |
| Matalascañas | M2020-47 | 19 |  |
| Matalascañas | M2020-58 | 20 |  |
| Matalascañas | M2020-68 | 22 |  |
| Matalascañas | M2020-84 | 19 |  |
| Matalascañas | M2020-85 | 20 |  |
| Matalascañas | M2020-89 | 21 |  |
| Matalascañas | M2020-92 | 20 |  |
| Le Rozel | LREI2016-10 | 18.4 | Duveau et al., 2020 |
| Le Rozel | LREI2016-11-12-13 | 12.3 |  |
| Le Rozel | LREI2013-05 | 21.4 |  |
| Le Rozel | LREI2015-43 | 24.9 |  |
| Le Rozel | LREI2016-71 | 23.6 |  |
| Le Rozel | LREI2016-72 | 17.5 |  |
| Le Rozel | LREI2016-79 | 22.1 |  |
| Le Rozel | LREI2015-04 | 11.4 |  |
| Le Rozel | LREI2015-46 | 18.5 |  |
| Le Rozel | LREI2015-47 | 18.3 |  |
| Le Rozel | LREI2015-49 | 16.2 |  |
| Le Rozel | LREI2015-53 | 18.0 |  |
| Le Rozel | LREI2016-03 | 13.8 |  |
| Le Rozel | LREI2016-25 | 16.2 |  |
| Le Rozel | LREI2016-33 | 16.1 |  |
| Le Rozel | LREI2016-35 | 21.5 |  |
| Le Rozel | LREI2016-36 | 13.1 |  |
| Le Rozel | LREI2016-39 | 16.9 |  |
| Le Rozel | LREI2016-56 | 16.4 |  |
| Le Rozel | LREI2016-60 | 14.8 |  |
| Le Rozel | LREI2017-07 | 15.4 |  |
| Le Rozel | LREI2017-13 | 17.9 |  |
| Le Rozel | LREI2017-18 | 19.2 |  |
| Le Rozel | LREI2017-22 | 20.5 |  |
| Le Rozel | LREI2017-32 | 21.3 |  |
| Le Rozel | LREI2017-41 | 18.1 |  |
| Le Rozel | LREI2017-47 | 24.7 |  |
| Le Rozel | LREI2017-49 | 21.4 |  |
| Le Rozel | LREI2017-52 | 23.2 |  |
| Le Rozel | LREI2017-56 | 22.9 |  |
| Le Rozel | LREI2017-57 | 19.4 |  |
| Le Rozel | LREI2017-64 | 23.4 |  |
| Le Rozel | LREI2017-71 | 23.4 |  |
| Le Rozel | LREI2017-73 | 22.1 |  |
| Le Rozel | LREI2017-75 | 17.3 |  |
| Le Rozel | LREI2017-86 | 28.4 |  |
| Le Rozel | LREI2017-87 | 27.5 |  |
| Le Rozel | LREI2017-95 | 18.8 |  |
| Le Rozel | LREI2017-101 | 15.8 |  |
| Le Rozel | LREI2017-102 | 16.4 |  |
| Le Rozel | LREI2017-103 | 22.1 |  |
| Le Rozel | LREI2017-104 | 17.0 |  |
| Le Rozel | LREI2017-105 | 21.7 |  |
| Le Rozel | LREI2017-110 | 19.3 |  |
| Le Rozel | LREI2017-111 | 16.9 |  |
| Le Rozel | LREI2017-112 | 15.6 |  |
| Le Rozel | LREI2016-07 | 16.4 |  |
| Le Rozel | LREI2016-18 | 17.5 |  |
| Le Rozel | LREI2016-19 | 26.8 |  |
| Le Rozel | LREI2016-61 | 17.5 |  |
| Le Rozel | LREI2016-66 | 19.5 |  |
| Le Rozel | LREI2016-67 | 15.0 |  |
| Le Rozel | LREI2016-68 | 21.7 |  |
| Le Rozel | LREI2016-73 | 22,8 |  |
| Theopetra | 2 | 15.1 | Manolis et al., 2000 |
| Theopetra | 3 | 13.8 |  |
| Vârtop |  | 22.0 | Onac et al., 2005 |
| Catalan Bay |  | 17.0 | Muñiz et al., 2019 |

**Table S7. Comparison of the width of the footprints of Matalascañas site with the Theopetra. Vârtop and Catalán Bay sites.**

| **Site** | **Inventory number** | **Width (cm)** | **References** |
| --- | --- | --- | --- |
| Matalascañas | M2020-01 | 6 | This article |
| Matalascañas | M2020-02 | 9 |  |
| Matalascañas | M2020-03 | 4 |  |
| Matalascañas | M2020-04 | 5 |  |
| Matalascañas | M2020-05 | 5 |  |
| Matalascañas | M2020-06 | 10 |  |
| Matalascañas | M2020-07 | 6 |  |
| Matalascañas | M2020-08 | 6 |  |
| Matalascañas | M2020-09 | 7 |  |
| Matalascañas | M2020-10 | 6 |  |
| Matalascañas | M2020-11 | 9 |  |
| Matalascañas | M2020-12 | 9 |  |
| Matalascañas | M2020-13 | 9 |  |
| Matalascañas | M2020-14 | 11 |  |
| Matalascañas | M2020-15 | 11 |  |
| Matalascañas | M2020-16 | 9 |  |
| Matalascañas | M2020-17 | 8 |  |
| Matalascañas | M2020-18 | 8 |  |
| Matalascañas | M2020-19 | 8 |  |
| Matalascañas | M2020-20 | 12 |  |
| Matalascañas | M2020-21 | 5 |  |
| Matalascañas | M2020-22 | 7 |  |
| Matalascañas | M2020-23 | 9 |  |
| Matalascañas | M2020-26 | 6 |  |
| Matalascañas | M2020-27 | 8 |  |
| Matalascañas | M2020-28 | 6 |  |
| Matalascañas | M2020-29 | 5 |  |
| Matalascañas | M2020-30 | 10 |  |
| Matalascañas | M2020-31 | 7 |  |
| Matalascañas | M2020-32 | 4 |  |
| Matalascañas | M2020-33 | 9 |  |
| Matalascañas | M2020-34 | 5 |  |
| Matalascañas | M2020-36 | 6 |  |
| Matalascañas | M2020-37 | 8 |  |
| Matalascañas | M2020-38 | 11 |  |
| Matalascañas | M2020-40 | 8 |  |
| Matalascañas | M2020-42 | 8 |  |
| Matalascañas | M2020-43 | 5 |  |
| Matalascañas | M2020-45 | 4 |  |
| Matalascañas | M2020-48 | 4 |  |
| Matalascañas | M2020-49 | 11 |  |
| Matalascañas | M2020-51 | 6 |  |
| Matalascañas | M2020-52 | 8 |  |
| Matalascañas | M2020-55 | 8 |  |
| Matalascañas | M2020-56 | 8 |  |
| Matalascañas | M2020-57 | 9 |  |
| Matalascañas | M2020-58 | 8 |  |
| Matalascañas | M2020-59 | 6 |  |
| Matalascañas | M2020-60 | 4 |  |
| Matalascañas | M2020-61 | 5 |  |
| Matalascañas | M2020-62 | 5 |  |
| Matalascañas | M2020-63 | 7 |  |
| Matalascañas | M2020-64 | 6 |  |
| Matalascañas | M2020-65 | 6 |  |
| Matalascañas | M2020-66 | 6 |  |
| Matalascañas | M2020-67 | 10 |  |
| Matalascañas | M2020-68 | 8 |  |
| Matalascañas | M2020-69 | 7 |  |
| Matalascañas | M2020-71 | 5 |  |
| Matalascañas | M2020-72 | 5 |  |
| Matalascañas | M2020-73 | 10 |  |
| Matalascañas | M2020-74 | 9 |  |
| Matalascañas | M2020-76 | 8 |  |
| Matalascañas | M2020-77 | 6 |  |
| Matalascañas | M2020-78 | 9 |  |
| Matalascañas | M2020-79 | 11 |  |
| Matalascañas | M2020-80 | 6 |  |
| Matalascañas | M2020-81 | 8 |  |
| Matalascañas | M2020-82 | 6 |  |
| Matalascañas | M2020-83 | 6 |  |
| Matalascañas | M2020-84 | 7 |  |
| Matalascañas | M2020-85 | 8 |  |
| Matalascañas | M2020-86 | 5 |  |
| Matalascañas | M2020-88 | 9 |  |
| Matalascañas | M2020-89 | 7 |  |
| Matalascañas | M2020-90 | 7 |  |
| Matalascañas | M2020-91 | 8 |  |
| Matalascañas | M2020-92 | 9 |  |
| Matalascañas | M2020-93 | 7 |  |
| Matalascañas | M2020-24 | 8 |  |
| Matalascañas | M2020-25 | 10 |  |
| Matalascañas | M2020-46 | 7 |  |
| Matalascañas | M2020-47 | 5 |  |
| Matalascañas | M2020-53 | 6 |  |
| Matalascañas | M2020-54 | 6 |  |
| Matalascañas | M2020-41 | 8 |  |
| Matalascañas | M2020-44 | 9 |  |
| Le Rozel | 2016-10 | 8.4 | Duveau et al., 2020 |
| Le Rozel | 2016-11-12-13 | 5.1 |  |
| Le Rozel | 2013-05 | 9.4 |  |
| Le Rozel | 2016-29 | 6.7 |  |
| Le Rozel | 2015-42 | 7.5 |  |
| Le Rozel | 2016-71 | 8.7 |  |
| Le Rozel | 2016-72 | 7.8 |  |
| Le Rozel | 2016-79 | 9.6 |  |
| Le Rozel | 2015-04 | 4.5 |  |
| Le Rozel | 2015-12 | 6.1 |  |
| Le Rozel | 2015-45 | 8.9 |  |
| Le Rozel | 2015-47 | 6.9 |  |
| Le Rozel | 2015-48 | 8.9 |  |
| Le Rozel | 2015-49 | 6.9 |  |
| Le Rozel | 2015-53 | 9.6 |  |
| Le Rozel | 2016-01 | 6.6 |  |
| Le Rozel | 2016-03 | 5.4 |  |
| Le Rozel | 2016-14 | 5.3 |  |
| Le Rozel | 2016-15 | 5.9 |  |
| Le Rozel | 2016-16 | 8.8 |  |
| Le Rozel | 2016-23 | 6.8 |  |
| Le Rozel | 2016-24 | 5.5 |  |
| Le Rozel | 2016-25 | 7.2 |  |
| Le Rozel | 2016-28 | 10.4 |  |
| Le Rozel | 2016-32 | 10.5 |  |
| Le Rozel | 2016-33 | 7.9 |  |
| Le Rozel | 2016-34 | 7.7 |  |
| Le Rozel | 2016-35 | 8.7 |  |
| Le Rozel | 2016-37 | 10.5 |  |
| Le Rozel | 2016-39 | 6.2 |  |
| Le Rozel | 2016-40;41 | 4.9 |  |
| Le Rozel | 2016-42 | 5.9 |  |
| Le Rozel | 2016-43 | 4.6 |  |
| Le Rozel | 2016-44 | 10.2 |  |
| Le Rozel | 2016-45 | 8.0 |  |
| Le Rozel | 2016-47 | 7.5 |  |
| Le Rozel | 2016-51 | 9.4 |  |
| Le Rozel | 2016-52 | 8.3 |  |
| Le Rozel | 2016-56 | 6.1 |  |
| Le Rozel | 2016-59 | 7.3 |  |
| Le Rozel | 2016-60 | 5.6 |  |
| Le Rozel | 2016-75;76 | 4.5 |  |
| Le Rozel | 2016-78 | 5.4 |  |
| Le Rozel | 2017-04 | 7.6 |  |
| Le Rozel | 2017-07 | 7.3 |  |
| Le Rozel | 2017-08 | 5.9 |  |
| Le Rozel | 2017-09 | 7.8 |  |
| Le Rozel | 2017-11 | 7.2 |  |
| Le Rozel | 2017-14 | 6.3 |  |
| Le Rozel | 2017-17 | 9.8 |  |
| Le Rozel | 2017-18 | 8.6 |  |
| Le Rozel | 2017-19 | 9.3 |  |
| Le Rozel | 2017-20 | 10.6 |  |
| Le Rozel | 2017-22 | 8.1 |  |
| Le Rozel | 2017-24 | 9.5 |  |
| Le Rozel | 2017-25 | 9.8 |  |
| Le Rozel | 2017-27 | 11.2 |  |
| Le Rozel | 2017-29 | 9.0 |  |
| Le Rozel | 2017-30 | 12.8 |  |
| Le Rozel | 2017-31 | 8.9 |  |
| Le Rozel | 2017-32 | 10.3 |  |
| Le Rozel | 2017-33 | 7.8 |  |
| Le Rozel | 2017-36 | 9.5 |  |
| Le Rozel | 2017-37 | 9.2 |  |
| Le Rozel | 2017-41 | 7.6 |  |
| Le Rozel | 2017-42 | 9.9 |  |
| Le Rozel | 2017-45 | 9.4 |  |
| Le Rozel | 2017-47 | 12.3 |  |
| Le Rozel | 2017-48 | 9.2 |  |
| Le Rozel | 2017-49 | 9.2 |  |
| Le Rozel | 2017-51 | 10.5 |  |
| Le Rozel | 2017-52 | 9.4 |  |
| Le Rozel | 2017-53 | 10.1 |  |
| Le Rozel | 2017-56 | 8.7 |  |
| Le Rozel | 2017-57 | 7.8 |  |
| Le Rozel | 2017-63 | 9.2 |  |
| Le Rozel | 2017-64 | 8.9 |  |
| Le Rozel | 2017-66 | 11.2 |  |
| Le Rozel | 2017-68 | 11.2 |  |
| Le Rozel | 2017-71 | 11.1 |  |
| Le Rozel | 2017-73 | 10.2 |  |
| Le Rozel | 2017-75 | 8.6 |  |
| Le Rozel | 2017-76 | 9.4 |  |
| Le Rozel | 2017-78 | 8.7 |  |
| Le Rozel | 2017-81 | 8.0 |  |
| Le Rozel | 2017-86 | 12.2 |  |
| Le Rozel | 2017-87 | 9.9 |  |
| Le Rozel | 2017-90 | 8.9 |  |
| Le Rozel | 2017-91 | 8.8 |  |
| Le Rozel | 2017-92 | 10.1 |  |
| Le Rozel | 2017-95 | 7.6 |  |
| Le Rozel | 2017-96 | 7.0 |  |
| Le Rozel | 2017-97 | 9.3 |  |
| Le Rozel | 2017-98 | 11.4 |  |
| Le Rozel | 2017-99 | 7.9 |  |
| Le Rozel | 2017-101 | 7.0 |  |
| Le Rozel | 2017-102 | 7.2 |  |
| Le Rozel | 2017-103 | 12.5 |  |
| Le Rozel | 2017-104 | 9.2 |  |
| Le Rozel | 2017-105 | 8.8 |  |
| Le Rozel | 2017-107 | 9.2 |  |
| Le Rozel | 2017-108 | 8.5 |  |
| Le Rozel | 2017-109 | 8.9 |  |
| Le Rozel | 2017-110 | 10.0 |  |
| Le Rozel | 2017-112 | 7.1 |  |
| Le Rozel | 2017-113 | 8.2 |  |
| Le Rozel | 2017-114 | 12.2 |  |
| Le Rozel | 2017-115 | 8.3 |  |
| Le Rozel | 2016-04 | 9.9 |  |
| Le Rozel | 2016-05 | 8.4 |  |
| Le Rozel | 2016-06 | 10.0 |  |
| Le Rozel | 2016-07 | 8.3 |  |
| Le Rozel | 2016-17 | 6.7 |  |
| Le Rozel | 2016-18 | 7.2 |  |
| Le Rozel | 2016-19 | 10.9 |  |
| Le Rozel | 2016-20 | 6.9 |  |
| Le Rozel | 2016-21 | 7.5 |  |
| Le Rozel | 2016-22 | 7.3 |  |
| Le Rozel | 2016-61 | 5.9 |  |
| Le Rozel | 2016-66 | 8.8 |  |
| Le Rozel | 2016-67 | 6.5 |  |
| Le Rozel | 2016-68 | 6.9 |  |
| Le Rozel | 2016-73 | 8.1 |  |
| Le Rozel | 2017-61 | 6.4 |  |
| Le Rozel | 2017-62 | 6.5 |  |
| Le Rozel | 2017-72 | 9.5 |  |
| Theopetra | 2 | 5.4 | Manolis et al., 2000 |
| Theopetra | 3 | 6.3 |  |
| Vârtop |  | 10.6 | Onac et al., 2005 |
| Catalan Bay |  | 7.0 | Muñiz et al., 2019 |

**Table S8. Comparison of the width / length ratio of the footprints of Matalascañas site with the Theopetra. Vârtop and Catalán Bay sites.**

| **Site** | **Inventory number** | **width / Length** | **References** |
| --- | --- | --- | --- |
| Matalascañas | M2020-01 | 0.30 | This article |
| Matalascañas | M2020-02 | 0.47 |  |
| Matalascañas | M2020-03 | 0.22 |  |
| Matalascañas | M2020-06 | 0.34 |  |
| Matalascañas | M2020-07 | 0.40 |  |
| Matalascañas | M2020-08 | 0.29 |  |
| Matalascañas | M2020-09 | 0.37 |  |
| Matalascañas | M2020-10 | 0.43 |  |
| Matalascañas | M2020-11 | 0.36 |  |
| Matalascañas | M2020-12 | 0.41 |  |
| Matalascañas | M2020-14 | 0.41 |  |
| Matalascañas | M2020-15 | 0.39 |  |
| Matalascañas | M2020-16 | 0.32 |  |
| Matalascañas | M2020-17 | 0.28 |  |
| Matalascañas | M2020-18 | 0.35 |  |
| Matalascañas | M2020-19 | 0.44 |  |
| Matalascañas | M2020-20 | 0.46 |  |
| Matalascañas | M2020-21 | 0.31 |  |
| Matalascañas | M2020-22 | 0.33 |  |
| Matalascañas | M2020-23 | 0,36 |  |
| Matalascañas | M2020-27 | 0.32 |  |
| Matalascañas | M2020-29 | 0.36 |  |
| Matalascañas | M2020-31 | 0.50 |  |
| Matalascañas | M2020-40 | 0.40 |  |
| Matalascañas | M2020-47 | 0.26 |  |
| Matalascañas | M2020-58 | 0.40 |  |
| Matalascañas | M2020-68 | 0.36 |  |
| Matalascañas | M2020-84 | 0.37 |  |
| Matalascañas | M2020-85 | 0.40 |  |
| Matalascañas | M2020-89 | 0.33 |  |
| Matalascañas | M2020-92 | 0.45 |  |
| Le Rozel | 2016-10 | 0.46 | Duveau et al., 2020 |
| Le Rozel | 2016-11-12-13 | 0.41 |  |
| Le Rozel | 2013-05 | 0.44 |  |
| Le Rozel | 2016-71 | 0.37 |  |
| Le Rozel | 2016-72 | 0.44 |  |
| Le Rozel | 2016-79 | 0.43 |  |
| Le Rozel | 2015-04 | 0.39 |  |
| Le Rozel | 2015-47 | 0.38 |  |
| Le Rozel | 2015-49 | 0.42 |  |
| Le Rozel | 2015-53 | 0.53 |  |
| Le Rozel | 2016-03 | 0.39 |  |
| Le Rozel | 2016-25 | 0.44 |  |
| Le Rozel | 2016-33 | 0.49 |  |
| Le Rozel | 2016-35 | 0.40 |  |
| Le Rozel | 2016-39 | 0.37 |  |
| Le Rozel | 2016-56 | 0.37 |  |
| Le Rozel | 2016-60 | 0.38 |  |
| Le Rozel | 2017-07 | 0.47 |  |
| Le Rozel | 2017-18 | 0.45 |  |
| Le Rozel | 2017-22 | 0.40 |  |
| Le Rozel | 2017-32 | 0.48 |  |
| Le Rozel | 2017-41 | 0.42 |  |
| Le Rozel | 2017-47 | 0.50 |  |
| Le Rozel | 2017-49 | 0.43 |  |
| Le Rozel | 2017-52 | 0.41 |  |
| Le Rozel | 2017-56 | 0.38 |  |
| Le Rozel | 2017-57 | 0.40 |  |
| Le Rozel | 2017-64 | 0.38 |  |
| Le Rozel | 2017-71 | 0.48 |  |
| Le Rozel | 2017-73 | 0.46 |  |
| Le Rozel | 2017-75 | 0.50 |  |
| Le Rozel | 2017-86 | 0.43 |  |
| Le Rozel | 2017-87 | 0.36 |  |
| Le Rozel | 2017-95 | 0.40 |  |
| Le Rozel | 2017-101 | 0.44 |  |
| Le Rozel | 2017-102 | 0.44 |  |
| Le Rozel | 2017-103 | 0.56 |  |
| Le Rozel | 2017-104 | 0.54 |  |
| Le Rozel | 2017-105 | 0.41 |  |
| Le Rozel | 2017-110 | 0.52 |  |
| Le Rozel | 2017-112 | 0.45 |  |
| Le Rozel | 2016-07 | 0.51 |  |
| Le Rozel | 2016-18 | 0.41 |  |
| Le Rozel | 2016-19 | 0.41 |  |
| Le Rozel | 2016-61 | 0.34 |  |
| Le Rozel | 2016-66 | 0.45 |  |
| Le Rozel | 2016-67 | 0.43 |  |
| Le Rozel | 2016-68 | 0.32 |  |
| Le Rozel | 2016-73 | 0.35 |  |
| Theopetra | 2 | 0.36 | Manolis et al., 2000 |
| Theopetra | 3 | 0.45 |  |
| Vârtop |  | 0.48 | Onac et al., 2005 |
| Catalan Bay |  | 0.41 | Muñiz et al., 2019 |

**Table S9.** Statures estimated from the total length of the footprints using the average of the estimates obtained from: 1) 15% ratio between foot length and stature. 2) the relationship used by Dingwall and his collaborators. and 3) that used by Duveau and his collaborators.

| **Inventory Number** | **Total length (cm)** | **Estimated statures (cm)** | | | |
| --- | --- | --- | --- | --- | --- |
|  |  | **15% ratio** | **Dingwall et al., 2013** | **Duveau et al., 2019** | **Mean** |
| M2020-01 | 20 | 133 | 149 | 130 | **137** |
| M2020-02 | 19 | 127 | 145 | 124 | **132** |
| M2020-03 | 18 | 120 | 141 | 117 | **126** |
| M2020-06 | 29 | 193 | 183 | 189 | **188** |
| M2020-07 | 15 | 100 | 130 | 98 | **109** |
| M2020-08 | 21 | 140 | 153 | 137 | **143** |
| M2020-09 | 19 | 127 | 145 | 124 | **132** |
| M2020-10 | 14 | 93 | 126 | 91 | **104** |
| M2020-11 | 25 | 167 | 168 | 163 | **166** |
| M2020-12 | 22 | 147 | 156 | 143 | **149** |
| M2020-14 | 27 | 180 | 175 | 176 | **177** |
| M2020-15 | 28 | 187 | 179 | 182 | **183** |
| M2020-16 | 28 | 187 | 179 | 182 | **183** |
| M2020-17 | 29 | 193 | 183 | 189 | **188** |
| M2020-18 | 23 | 153 | 160 | 150 | **154** |
| M2020-19 | 18 | 120 | 141 | 117 | **126** |
| M2020-20 | 26 | 173 | 172 | 169 | **171** |
| M2020-21 | 16 | 107 | 134 | 104 | **115** |
| M2020-22 | 21 | 140 | 153 | 137 | **143** |
| M2020-23 | 25 | 167 | 168 | 163 | **166** |
| M2020-27 | 25 | 167 | 168 | 163 | **166** |
| M2020-29 | 14 | 93 | 126 | 91 | **104** |
| M2020-31 | 14 | 93 | 126 | 91 | **104** |
| M2020-40 | 20 | 133 | 149 | 130 | **137** |
| M2020-47 | 19 | 127 | 145 | 124 | **132** |
| M2020-58 | 20 | 133 | 149 | 130 | **137** |
| M2020-68 | 22 | 147 | 156 | 143 | **149** |
| M2020-84 | 19 | 127 | 145 | 124 | **132** |
| M2020-85 | 20 | 133 | 149 | 130 | **137** |
| M2020-89 | 21 | 140 | 153 | 137 | **143** |
| M2020-92 | 20 | 133 | 149 | 130 | **137** |
